# Supplementary material for: Tree representations of brain structural connectivity via persistent homology
Source: Front Neurosci. 2023 Oct 13;17:1200373. doi: 10.3389/fnins.2023.1200373 (PMC10603366; doi:10.3389/fnins.2023.1200373)
Supplement: Supplementary file 1 [file Data_Sheet_1.pdf]

# Supplementary Material for “Tree Representations of Brain Structural Connectivity via Persistent Homology”

**Didong Li** [1,*,†], **Phuc Nguyen** [2,†], **Zhengwu Zhang** [3] **and David Dunson** [2]

[1] *Department of Biostatistics, University of North Carolina at Chapel Hill, Chapel Hill, NC, USA.*
[2] *Department of Statistial Science, Duke University, Durham, NC, USA.*
[3] *Department of Statistics and Operation Research, University of North Carolina at Chapel Hill, Chapel Hill, NC, USA.*
† *These authors contributed equally to this work and share first authorship.*

Correspondence*:
Corresponding Author
didongli@unc.edu

## 1 HIERARCHY IN ONE HEMISPHERE BASED ON THE DESIKAN-KILLIANY PROTOCOL

Figure 1 shows the hierarchy in one hemisphere based on the DK protocol.

## 2 PROOF OF THEOREM IN SECTION 2

We first restate Theorem **??** in topological language. Let $x_j^l \in A_j^l$ be any location in brain region $A_j^l$ and any fiber inside $A_j^l$ is treated as a loop at $x_j^l$, denoted by $c_{j(1)}^l, \cdots, c_{j(N_j^l)}^l$. So topologically speaking each region $A_j^l$ consists of a point (0-cell) and $N_j^l$ loops (1-cell). Assume $A_i^l$ and $A_j^l$ are children of $A_k^{l-1}$, then we have a inclusion map from the children to the parent: $F_k^l : A_i^l \cup A_j^l \to A_k^{l-1}$, $F(x_j^l) = F(x_i^l) = x_k^{l-1}$, so $F_k^l$ induces a homomorphism between chain groups: $F_k^l : C_p(A_i^l \cup A_j^l) \to C_p(A_k^{l-1})$, $p = 0, 1$. Observe that $F$ maps all loops $c_{j(k)}^l$ and $c_{i(k)}^l$ to loops in $A_k^{l-1}$ while any fiber (path) connecting $A_i^l$ and $A_j^l$ is also mapped to loops in $A_k^{l-1}$.

THEOREM **??**'. *$H_k^l$ is the corank of the persistent homology* $F_{k*}^l \left( H_p(A_i^l \cup A_j^l) \right) \subset H_p(A_k^{l-1})$.

Before proving the theorem, we prove the following useful lemma:

LEMMA 1. *$F$ defined above is a chain map so it induces a homomorphism between homology groups:*

$$F_{k*}^l : H_p(A_i^l \cup A_j^l) \to H_p(A_k^{l-1}),\ p = 0, 1.$$

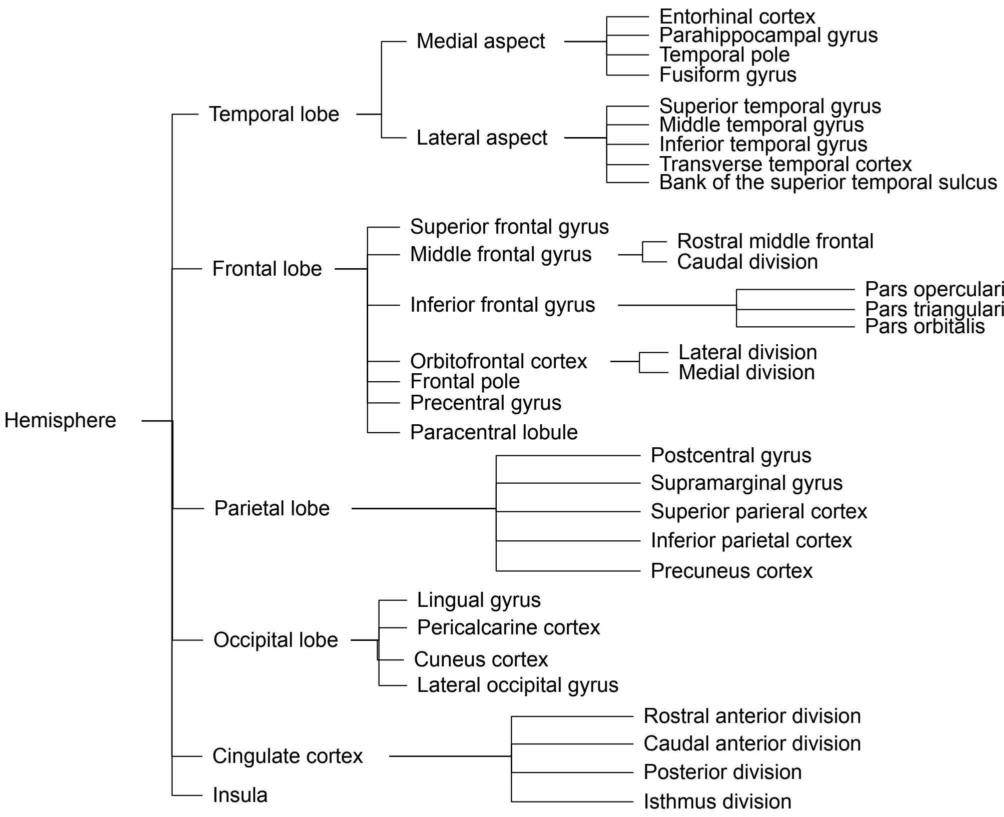


**Figure 1.** The full brain hierarchy based on the Desikan-Killiany protocol for one hemisphere.

PROOF. Since the chain group is nontrivial for $p = 0$ and $p = 1$ only, it suffices to check $\partial \circ F_k^l(c) = F_k^l \circ \partial(c)$ for any $c \in C_1(A_i^l \cup A_j^l)$.

If $c$ is a loop, then $F_k^l(c)$ is also a loop by the construction of $F_k^l$, so we have $\partial \circ F(c) = x_k^{l-1} - x_k^{l-1} = 0$, $F_k^l \circ \partial(c) = F_k^l(0) = 0$.

If $c$ is not a loop, we assume $c$ starts from $x_j^l$ ends at $x_i^l$, so $F_k^l(c)$ is still a loop, so we have $\partial \circ F_k^l(c) = x_k^{l-1} - x_k^{l-1} = 0$. Since $F_k^l \circ \partial(c) = F_k^l(x_i^l - x_j^l) = x_k^{l-1} - x_k^{l-1} = 0$, we conclude that $\partial \circ F_k^l = F_k^l \circ \partial$.

Now we can prove Theorem **??**':

PROOF OF THEOREM **??**'. From the proof of Lemma 1, $\text{rank}(H_p(A_k^{l-1}))$ is the sum of the number of fibers in $A_i^l$ and $A_j^l$ as well as the number of fibers connecting $A_i^l$ and $A_j^l$. Since all homologous classes in $H_p(A_i^l \cup A_j^l)$ are mapped to nontrivial homologous classes in $H_p(A_k^{l-1})$, $\text{rank}\left(F_{k*}^l\left(H_p(A_i^l \cup A_j^l)\right)\right) = \text{rank}\left(H_p(A_i^l \cup A_j^l)\right)$, which is equal to the number of fibers in fibers in $A_i^l$ and $A_j^l$. As a result,

$$\text{corank}\left(F_{k*}^l\left(H_p(A_i^l \cup A_j^l)\right)\right) = \text{rank}(H_p(A_k^{l-1})) - \text{rank}\left(F_{k*}^l\left(H_p(A_i^l \cup A_j^l)\right)\right) = H_k^l.$$

## 3 TABLE OF 45 TRAITS USED IN SECTION 3.2 ANALYSIS

Table 1 lists the different traits used in our analyses grouped into categories; we provide the names used in the HCP data files along with a link to look up a detailed definition of each of these traits.

| Trait category | HCP column names |
|---|---|
| Cognition | PMAT24_A_CR, ReadEng_AgeAdj, PicVocab_AgeAdj, IWRD_TOT, ProcSpeed_AgeAdj, DDisc_AUC_200, DDisc_AUC_40K, VSPLOT_TC, SCPT_TPRT, ListSort_AgeAdj, PicSeq_AgeAdj, SSAGA_Educ, SSAGA_Income, CardSort_AgeAdj, Flanker_AgeAdj |
| Emotion | ER40_CRT, AngAffect_Unadj, AngHostil_Unadj, AngAggr_Unadj, FearAffect_Unadj, FearSomat_Unadj, Sadness_Unadj, PercStress_Unadj, SelfEff_Unadj, LifeSatisf_Unadj, MeanPurp_Unadj, PosAffect_Unadj |
| Tobacco use | SSAGA_TB_Age_1st_Cig, SSAGA_TB_DSM_Difficulty_Quitting, SSAGA_TB_Max_Cigs, SSAGA_TB_Reg_CPD, SSAGA_TB_Yrs_Smoked, Times_Used_Any_Tobacco_Today, Avg_Weekend_Any_Tobacco_7days, Total_Cigars_7days, Avg_Weekend_Cigars_7days, Num_Days_Used_Any_Tobacco_7days |
| Drug use | SSAGA_Times_Used_Illicits, SSAGA_Times_Used_Cocaine, SSAGA_Times_Used_Hallucinogens, SSAGA_Times_Used_Opiates, SSAGA_Times_Used_Sedatives, SSAGA_Times_Used_Stimulants, SSAGA_Mj_Age_1st_Use, SSAGA_Mj_Times_Used |

**Table 1.** Column names from the HCP data file of traits used in our analysis, grouped by categories defined by their meanings. Since there are many metrics for the same trait, we provide the column names so that their exact definitions can be looked up at `https://wiki.humanconnectome.org/display/PublicData/HCP-YA+Data+Dictionary-+Updated+for+the+1200+Subject+Release`.

## 4 SENSITIVITY ANALYSIS USING THREE-LEVEL TREE REPRESENTATION

We performed the same three analyses as in sections 3.2, 3.3, 3.4 but for a three-level tree representation and observed that the results were still comparable to the adjacency matrix representation. Results of this sensitivity analysis are shown in Figure 2, 3, 4, and 5. Therefore, the conclusion in our paper remains.

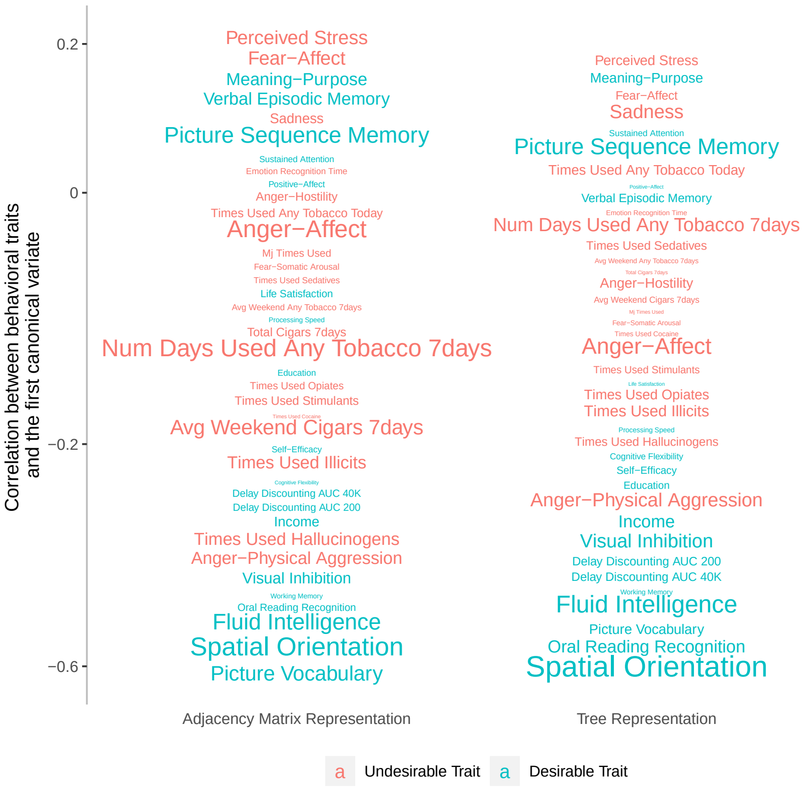


**Figure 2.** Correlations between behavioral traits and the first canonical variate extracted from 13 principal components of the AM compared to the 13 non-leaf nodes of the three-level tree representation. The y-axis has been transformed so that traits do not overlap. The font size of each trait indicates the magnitude of the coefficients of a linear combination that defines the first canonical variate. Results are similar to those of the four-level DK tree in Figure **??**.

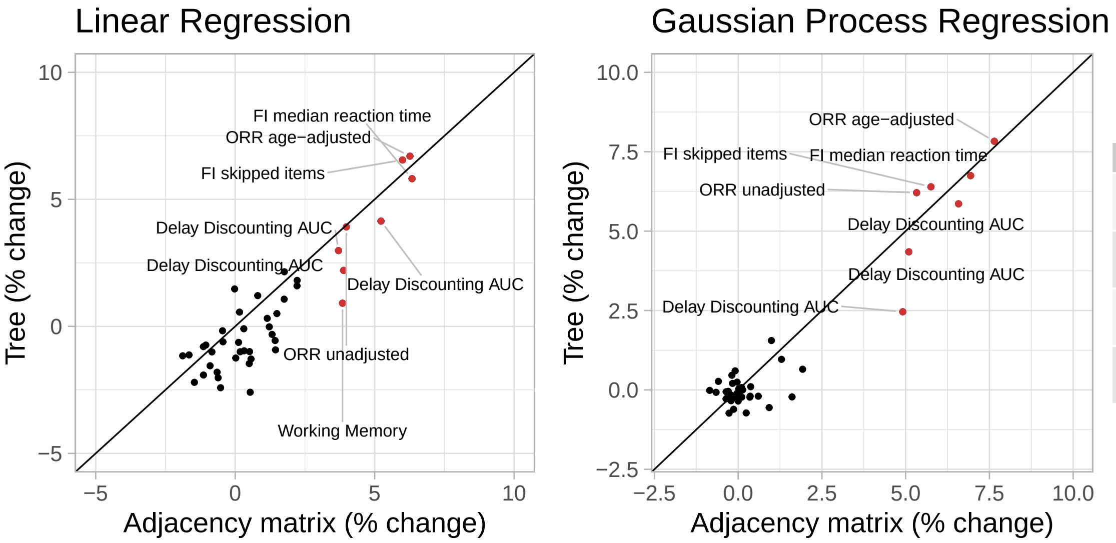


| acronym | trait |
|---|---|
| FI | Fluid intelligence |
| ORR | Oral reading recognition |
| PV | Picture vocabulary |
| SO | Spatial orientation |

**Figure 3.** Percentage of change in MSE compared to baseline of linear regression and GP regression using three-level tree and 13 PCs of the AM representation in predicting 45 cognitive traits

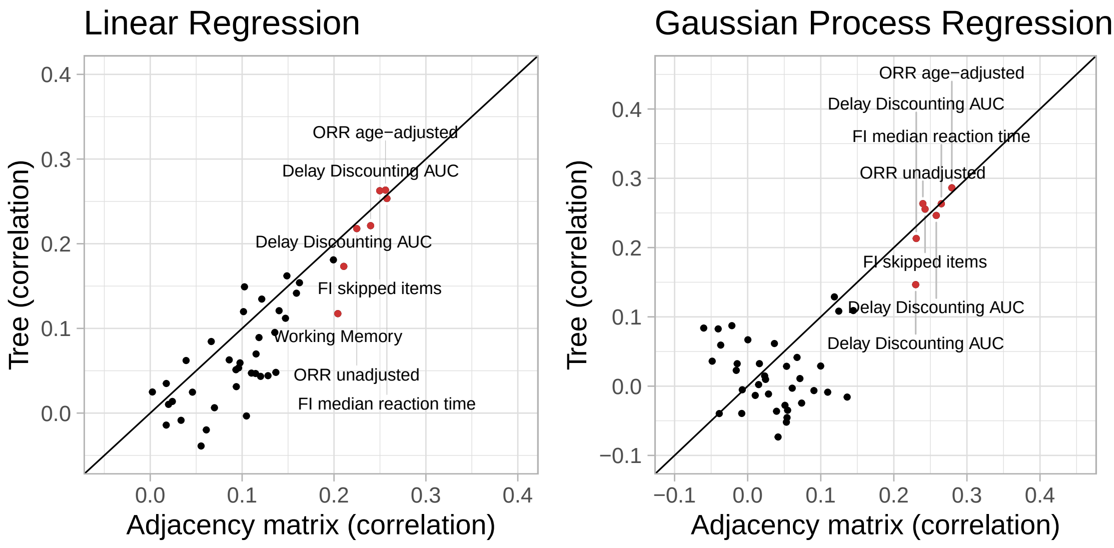


| acronym | trait |
|---|---|
| FI | Fluid intelligence |
| ORR | Oral reading recognition |
| PV | Picture vocabulary |
| SO | Spatial orientation |

**Figure 4.** Correlation between predictions and observed outcomes of linear regression and GP regression using three-level tree and 13 PCs of AM representation in predicting 45 cognitive traits

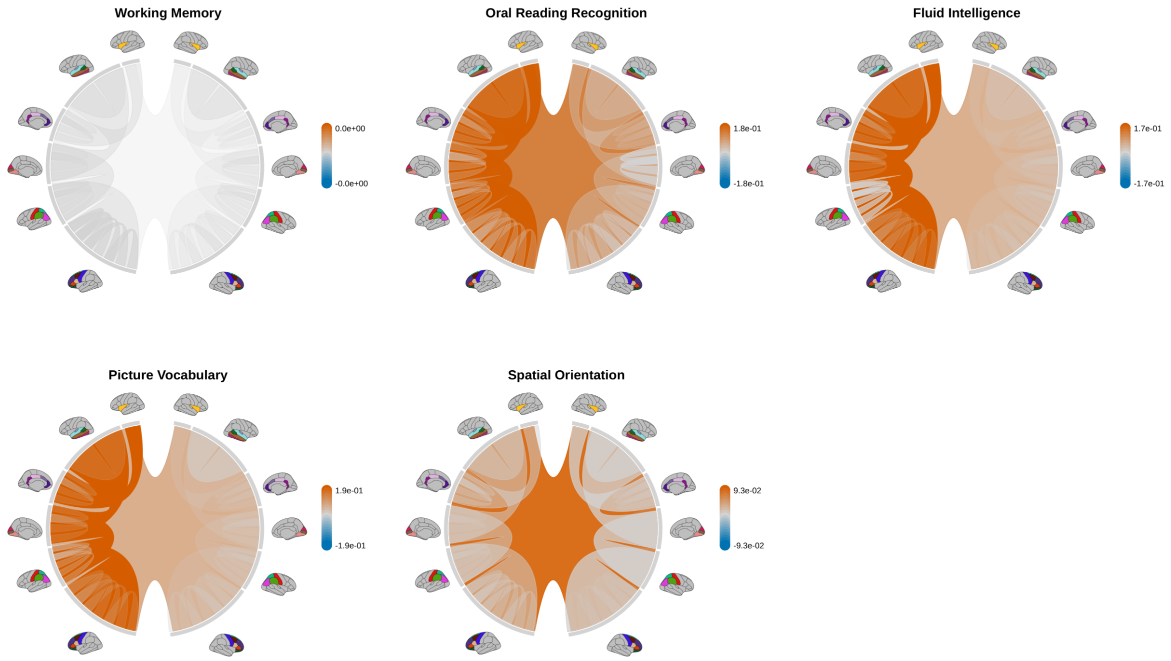


**Figure 5.** Brain connectome structures significantly associated with each trait inferred using the three-level tree representation. Colors represent the sign and magnitude of significant (i.e., posterior inclusion probability greater than 0.75) regression coefficients. For tree-based results, the opacity also represents the posterior inclusion probability. Results are similar to those of the four-level DK tree in Figure **??** and **??**.

## 5 VISUALIZATION OF RESULTS FROM SECTION 3.4 AS TREE PLOTS

We present alternative visualizations of the tree representations results in Figure **??** (left) and **??** (left). Instead of the circle chord plot, we visualize the full tree structure (excluding leaf nodes), brain regions

included in each tree node, and associations between each node and the trait of interest. Black dots represent DK regions within a tree node. The colors of the edges between dots represent the sign and magnitude of significant (i.e., posterior inclusion probability greater than 0.75) regression coefficients. Insignificant regression coefficients are equivalent to zero.

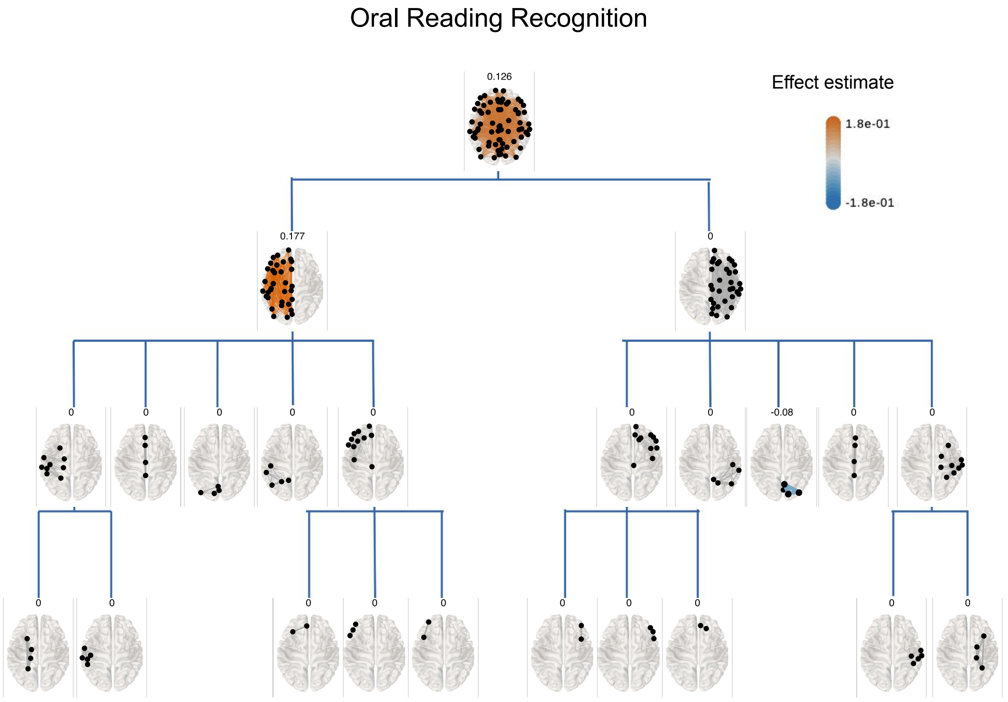


**Figure 6.** Brain connectome structures significantly associated with oral reading recognition inferred using the DK tree representation.

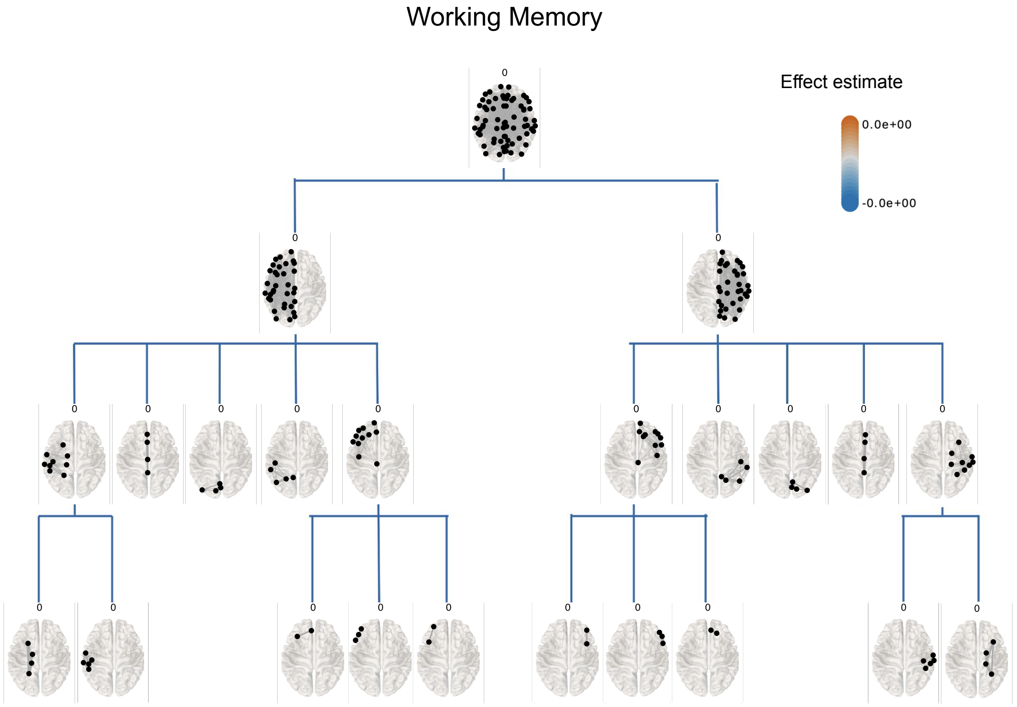


**Figure 7.** Brain connectome structures significantly associated with working memory inferred using the DK tree representation.

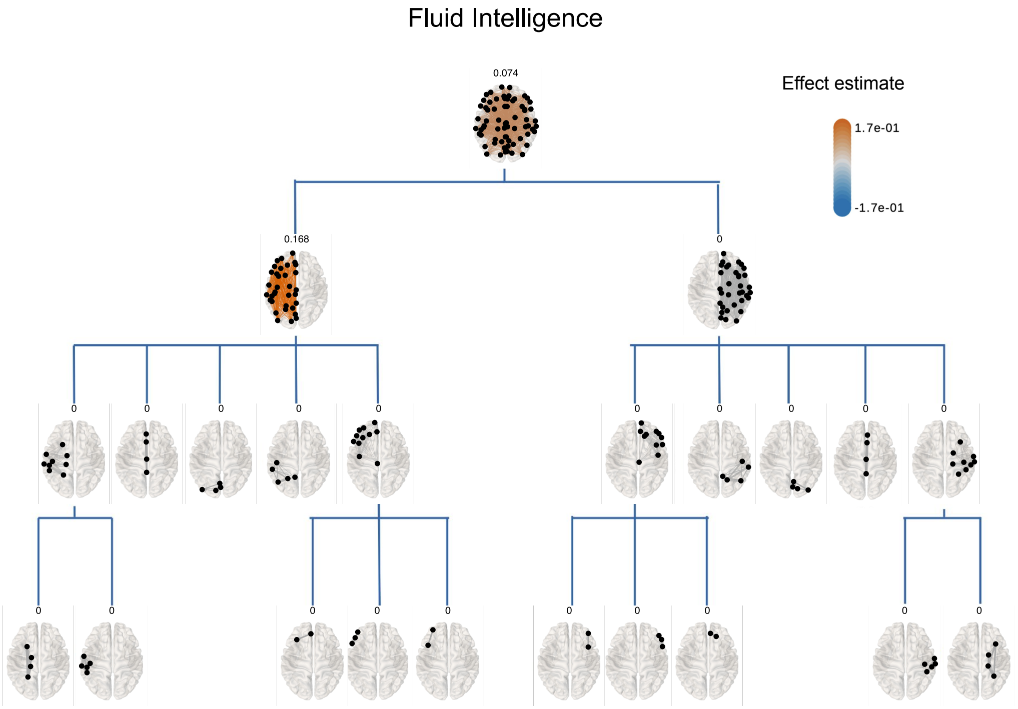


**Figure 8.** Brain connectome structures significantly associated with fluid intelligence inferred using the DK tree representation.

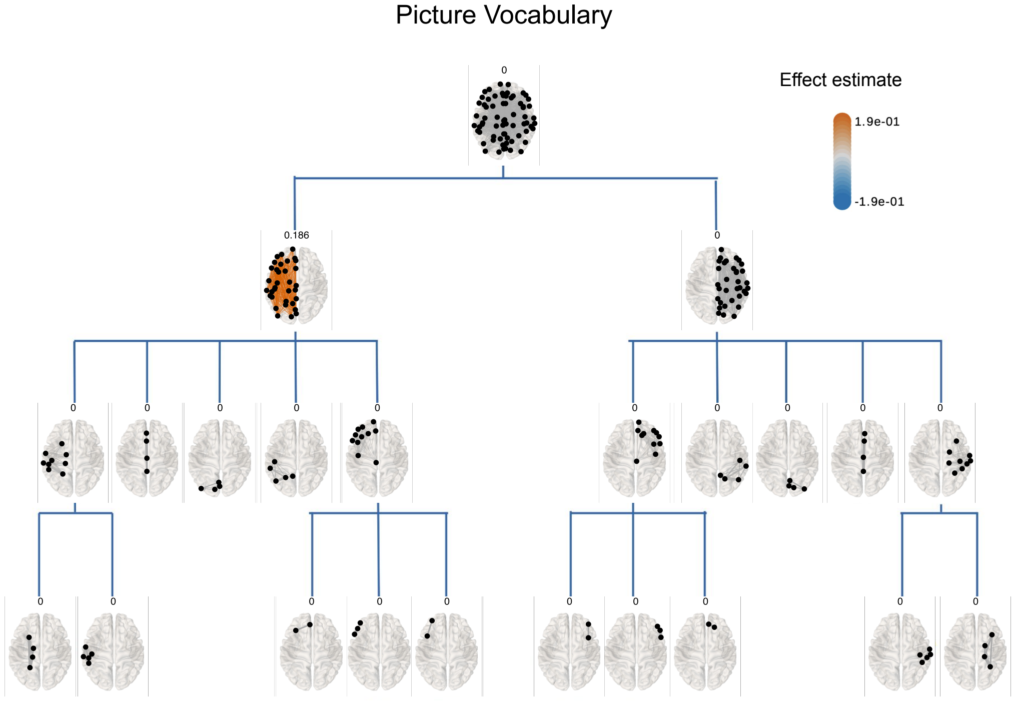


**Figure 9.** Brain connectome structures significantly associated with picture vocabulary inferred using the DK tree representation.

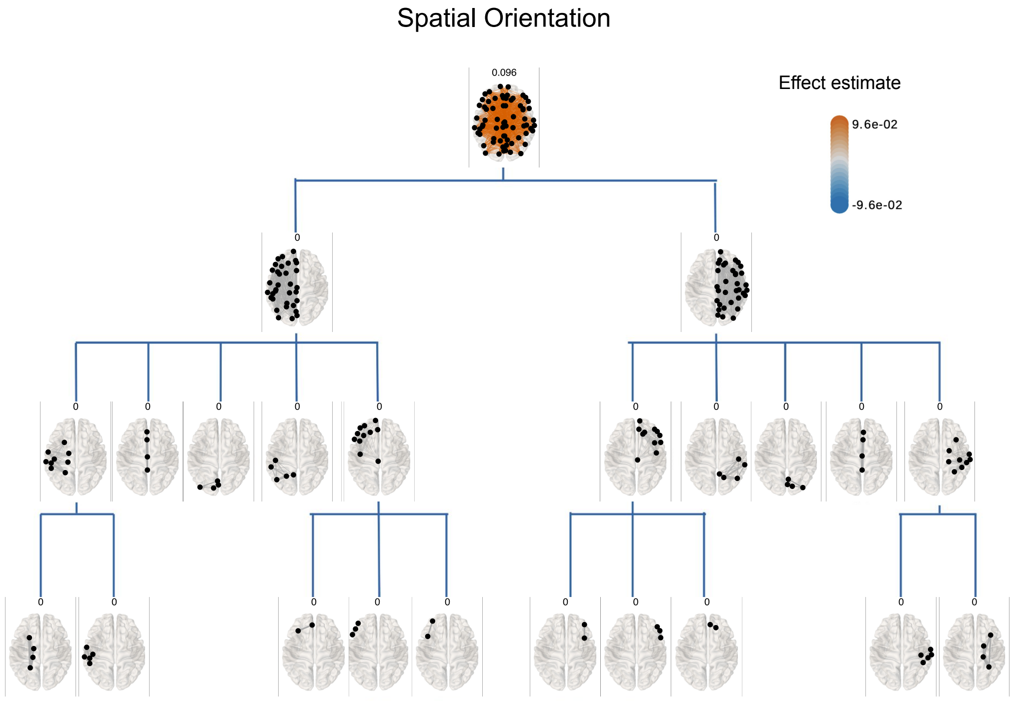


**Figure 10.** Brain connectome structures significantly associated with spatial orientation inferred using the DK tree representation.
